# Computed tomography-based thermography (CTT) in microwave ablation: prediction of the heat ablation zone in the porcine liver

**DOI:** 10.1186/s13244-023-01537-z

**Published:** 2023-11-14

**Authors:** Bogdan Kostyrko, Kerstin Rubarth, Christian Althoff, Franz Gerd Martin Poch, Christina Ann Neizert, Miriam Zibell, Bernhard Gebauer, Kai Siegfried Lehmann, Stefan Markus Niehues, Jürgen Mews, Torsten Diekhoff, Julian Pohlan

**Affiliations:** 1 Department of Radiology, Freie Universität Berlin and Humboldt-Universität Zu Berlin, Charitéplatz 1, 10117 Berlin, Germany; 2 Institute of Biometry and Clinical Epidemiology, Freie Universität Berlin and Humboldt-Universität Zu Berlin, Charitéplatz 1, 10117 Berlin, Germany; 3 Institute of Medical Informatics, Freie Universität Berlin and Humboldt-Universität Zu Berlin, Charitéplatz 1, 10117 Berlin, Germany; 4 Department of General and Visceral Surgery, Freie Universität Berlin and Humboldt-Universität Zu Berlin, Hindenburgdamm 30, 12200 Berlin, Germany; 5 Canon Medical Systems Europe BV, Global Research & Development Center, Amstelveen, the Netherlands; 6 Berlin Institute of Health at Charité – Universitätsmedizin Berlin, Charitéplatz 1, 10117 Berlin, Germany

**Keywords:** Computed tomography, Microwave ablation, Ablation zone prediction, In vivo experiment, Computed tomography thermography

## Abstract

**Objectives:**

The aim of the study was to investigate computed tomography-based thermography (CTT) for ablation zone prediction in microwave ablation (MWA).

**Methods:**

CTT was investigated during MWA in an in vivo porcine liver. For CTT, serial volume scans were acquired every 30 s during ablations and every 60 s immediately after MWA. After the procedure, contrast-enhanced computed tomography (CECT) was performed. After euthanasia, the liver was removed for sampling and further examination. Color-coded CTT maps were created for visualization of ablation zones, which were compared with both CECT and macroscopy. Average CT attenuation values in Hounsfield units (HU) were statistically correlated with temperatures using Spearman’s correlation coefficient. CTT was retrospectively evaluated in one patient who underwent radiofrequency ablation (RFA) treatment of renal cell carcinoma.

**Results:**

A significant correlation between HU and temperature was found with *r* =  − 0.77 (95% confidence interval (CI), − 0.89 to − 0.57) and *p* < 0.001. Linear regression yielded a slope of − 1.96 HU/°C (95% CI, − 2.66 to − 1.26). Color-coded CTT maps provided superior visualization of ablation zones.

**Conclusion:**

Our results show that CTT allows visualization of the ablation area and measurement of its size and is feasible in patients, encouraging further exploration in a clinical setting.

**Critical relevance statement:**

CT-based thermography research software allows visualization of the ablation zone and is feasible in patients, encouraging further exploration in a clinical setting to assess risk reduction of local recurrence.

**Graphical Abstract:**

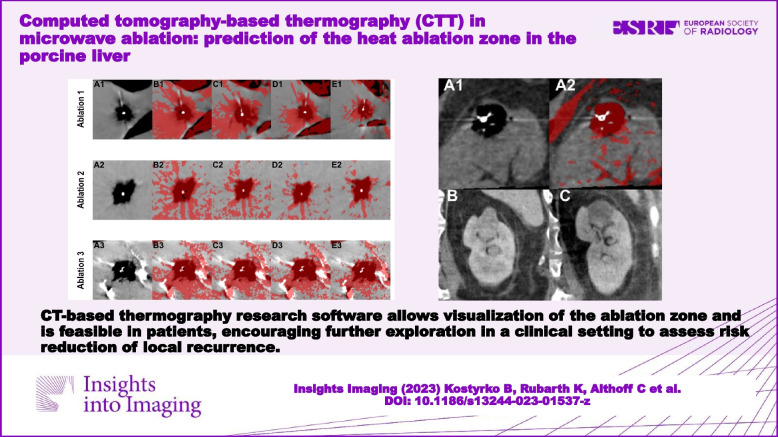

**Supplementary Information:**

The online version contains supplementary material available at 10.1186/s13244-023-01537-z.

## Key points

• Intraprocedural ablation monitoring is important for risk reduction of local recurrence.

• CT-based thermography is used for noninvasive ablation zone monitoring.

• CTT visualized ablated area to evaluate the success of the ablation.

**Supplementary Information:**

The online version contains supplementary material available at 10.1186/s13244-023-01537-z.

## Introduction

Thermal ablation is commonly used for local tumor treatment as it induces tissue necrosis by applying heat or cold [1, 2]. To avoid local recurrence, which is the main problem of the method, intraprocedural ablation monitoring is essential [3]. To stimulate cell death in the defined area by heating, the temperature must reach at least 60 °C [4]. Radiofrequency ablation (RFA) and microwave ablation (MWA) are two common heat ablation techniques with different advantages and disadvantages. Unlike other thermal ablation techniques, MWA creates an ablation zone that is characterized by a sharp boundary and takes on a round shape with the smallest possible periablational zone-to-coagulation zone ratio [5, 6]. In the ablation zone, target tissue coagulation is induced by heat created around the microwave probe [5, 6].

After thermal ablation, three zones with different cell death rates can be defined histologically with increasing distance from the probe [7, 8]. The zone immediately adjacent to the ablation probe is known as the white zone and is characterized by complete tissue necrosis and instant loss of vital cells [8]. This necrotic zone is surrounded by a light red zone that contains damaged cells and also living vital cells [8]. However, cells in this area will necrotize over time, which is why this area is considered to be part of the ablation zone [7, 9]. The outer red zone with intercellular edema contains vital cells and forms a transition area to healthy tissue [7, 8]. This zone should not be considered when assessing ablation success. Following an ablation procedure, contrast-enhanced computed tomography (CECT) or magnetic resonance imaging (MRI) is commonly used as the gold standard to assess ablation success [10]. For optimal assessment of outcome, the same imaging modality should be used during and after the intervention to ensure optimal image registration [10]. In our study, we used CECT. However, CECT may overestimate the necrotic zone, as the transition zone might retain living tumor cells but will show similarly low contrast uptake as the white zone [7]. Thus, CECT cannot reliably visualize the extent of the necrotic area [7]. So far, the success of ablation can only be assessed after treatment, as noninvasive intraprocedural temperature measurement is currently unavailable.

The potential of computed tomography (CT)-based thermography (CTT) for ablation zone monitoring has been investigated in ex vivo and in vivo [3, 11, 12]. This imaging method is based on an inverse correlation between Hounsfield units (HU) in CT scans and the temperature within the ablation area. Heat development during thermal ablation causes tissue attenuation to decrease, thereby demarcating the ablation zone on CT. With increasing distance from the probe, the change in HU decreases and approaches the initial value in thermally unaltered tissue [13]. Because of different experimental setups or ablated tissues, measured HU/temperature slopes reported for CTT in the literature are not consistent [3, 14].

The aim of this study was to investigate CTT for MWA zone prediction comparing volume CT datasets with CECT findings and macroscopic assessment in an in vivo porcine liver model.

## Methods

### Animal care and housing

A healthy 4-month-old female domestic pig under general anesthesia was used for this experiment. The pig was housed in the central animal facility of the Charité following the 2010/63/EU guidelines as well as the recommendation of the GM-Solas (Gesellschaft für Versuchstierkunde, Freiburg, Germany). At the end of the experiment, the pig was euthanized under deep general anesthesia.

### Experimental setup

A total of three MWAs were performed in the healthy pig liver and monitored with CT. During each MWA, the ablation probe (Emprint System, Medtronic, Meerbusch, Germany) was set to a power of 100 Watt, and ablation was performed for 5 min, as commonly done in clinical practice [15, 16]. The probe was placed in the liver, and two custom-made fiberoptic thermometers (Optocon and Weidmann Technologies, Dresden, Germany) were inserted parallel to the probe for continuous temperature monitoring during the procedure. Individual ablation areas were placed sufficient distance from each other. At the end of the experiment, the liver was removed for sampling and further examination. After liver resection, the ablation lesions were bisected and examined macroscopically (Supplementary Fig. 1, A1–A3). The samples were photographed on a sheet of scaled millimeter paper, which allows precise sizing of the ablation area with dedicated software (MWANecrosisMeasurement, Fraunhofer Institute for Digital Medicine MEVIS, Bremen, Germany) [17].

### Computed tomography protocol

Each MWA was monitored by CT, for which 20 spectral scans were acquired without table movement using 16-cm detector coverage (Canon Aquilion ONE Prism; Canon Medical Systems, Otawara, Japan). The following scan parameters were used: rapid kVp switching between 80 and 135 kVp, 1 s rotation time, and 500 mA tube current. The first ten scans were acquired every 30 s beginning before ablation started (*T*_0_) and ending with the time of peak temperature (*T*_max_) to cover the upslope phase. The next 10 scans were acquired at 60-s intervals and covered the downslope, postablation phase. After withdrawal of the MWA probe, a CECT scan in the portal venous phase following administration of an intravenous (IV) contrast agent (100 ml Imeron 400 MCT, Bracco, Konstanz) was acquired for each ablation (Supplementary Fig. 1, B1–B3). CECT was performed according to a previously established protocol with a fixed amount of contrast medium [7, 18].

### Image reconstruction and registration

The CT system generated virtual mono-energetic reconstructions in a medium soft-tissue kernel using Advanced Intelligent Clear-IQ Engine (spectral AICE) with 75 keV. Primary reconstructions resulted in 0.5-mm slice thickness volume stacks. Motion artifacts were reduced by applying a double-registration approach consisting of elastic registration followed by rigid registration, selecting the probe tip at the time of maximum temperature in the center of the ablation zone as the reference point [19].

### Computed tomography thermography

**Fig. 1:**
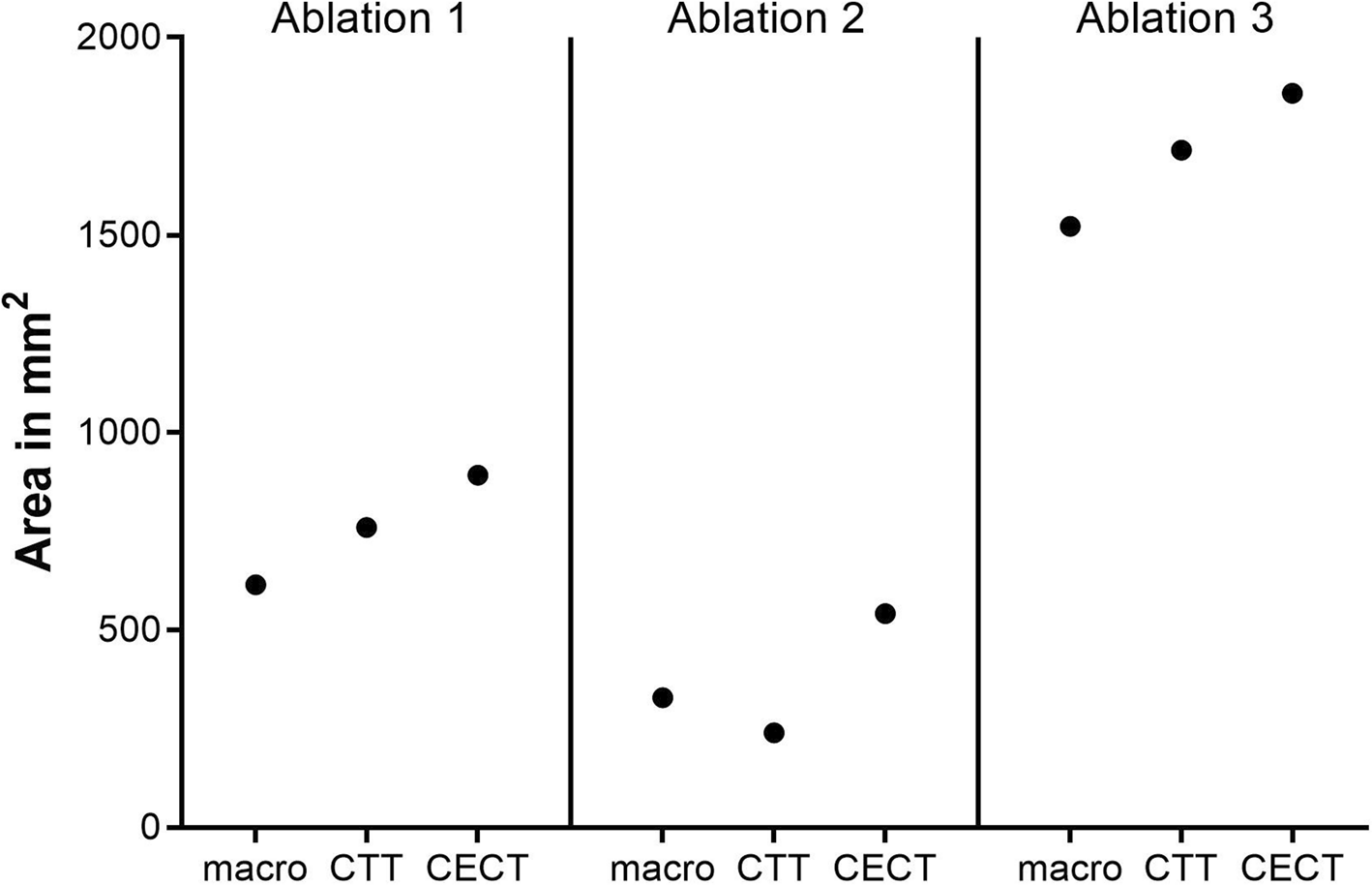
Ablation areas determined by macroscopy and two CT imaging modalities. macro, macroscopy; CTT, computed tomography thermography; area measured using the slope of − 1.96 HU/°C. CECT, contrast-enhanced computed tomography

Based on macroscopic measurement, circular ROIs were defined specifically for each ablation to determine average attenuation (HU) in unenhanced CT scans during the upslope phase. These ROIs exceeded the largest macroscopically measured diameter by a few millimeters and were determined as follows: ablation A—28 + 2 mm, ablation B—22 + 2 mm, ablation C—44 + 2 mm (data presented as mean value of two measured diameters on each side of the lesion after bisection + small extension zone of 2 mm). These data and invasively measured temperatures served to calculate the slope using pooled data of all three ablations. A dedicated research CTT software (Canon Medical Systems, Japan) was used to visualize the ablation area. Here, (at least) two CT scans must be uploaded: one scan from before ablation (*T*_0_) and another from the time of peak temperature (*T*_max_). To create a thermography map after uploading the registered CTT dataset, a slope is required, which was calculated before. The result is a colored map superimposed on the uploaded CT scan at *T*_max_. Red was chosen for better visualization of the ablated area and represents the temperature changes (ΔT) in the tissue. For the present study, the sensitivity of the colored map was set to indicate temperature changes greater than 33 K (ΔT ≥ 33 K). Thus, it can be assumed that at a core body temperature of 37 °C, tissue necrosis has been achieved at 70 °C within the colored zone (*T*_core_ + ΔT > 70 °C). The largest extent of the ablation area in CTT was measured in the para-axial plane, perpendicular to the inserted probe. The ablation areas determined using CTT were compared with macroscopic size measurements and the ablation areas identified by CECT (Fig. 1). The ablation zone in CECT can be detected orthogonal to the probe insertion region. It is characterized by a lower tissue density with an enhancing margin, whereas a differentiated distinction of histological zones is not possible [7, 10].

### Retrospective clinical analysis

A patient with unresectable renal cell carcinoma (RCC) at the left renal pole was selected for retrospective analysis of the potential of CTT in a clinical setting. The patient underwent RFA of the RCC over 10 min using the RITA Starburst Semi-Flex applicator (Rita Medical Systems, Milwaukee, WI, USA). Pre- and postablation CT scans were acquired using the same acquisition parameters. The two scans were then selected to retrospectively determine the ablation zone based on CTT.

### Statistics

Average HU values determined in the ROIs placed in unenhanced CT scans were matched with the corresponding temperatures measured invasively during the upslope phase. Spearman’s correlation coefficient was calculated after performing data normality tests, and linear regression was performed for pooled data of all three ablations in the upslope phase. All statistics were planned in consultation with a biometric expert. Calculations were carried out using GraphPad Prism (Version 9.5.1, San Diego, California) software. A *p*-value < 0.05 was considered statistically significant.

## Results

In this experiment, a total of 60 CT scans were successfully acquired with an average CTDIvol (CT dose index-volume) of 20.8 mGy. Use of a 16-cm detector yielded an average DLP (dose-length product) of 332.8 mGy per scan. Although the same MWA probe energy power was used for all three ablations, the sizes of the resulting ablation zones determined by macroscopic assessment varied: 615 mm^2^, 329 mm^2^, and 1522 mm^2^ (Fig. 1).

**Fig. 2:**
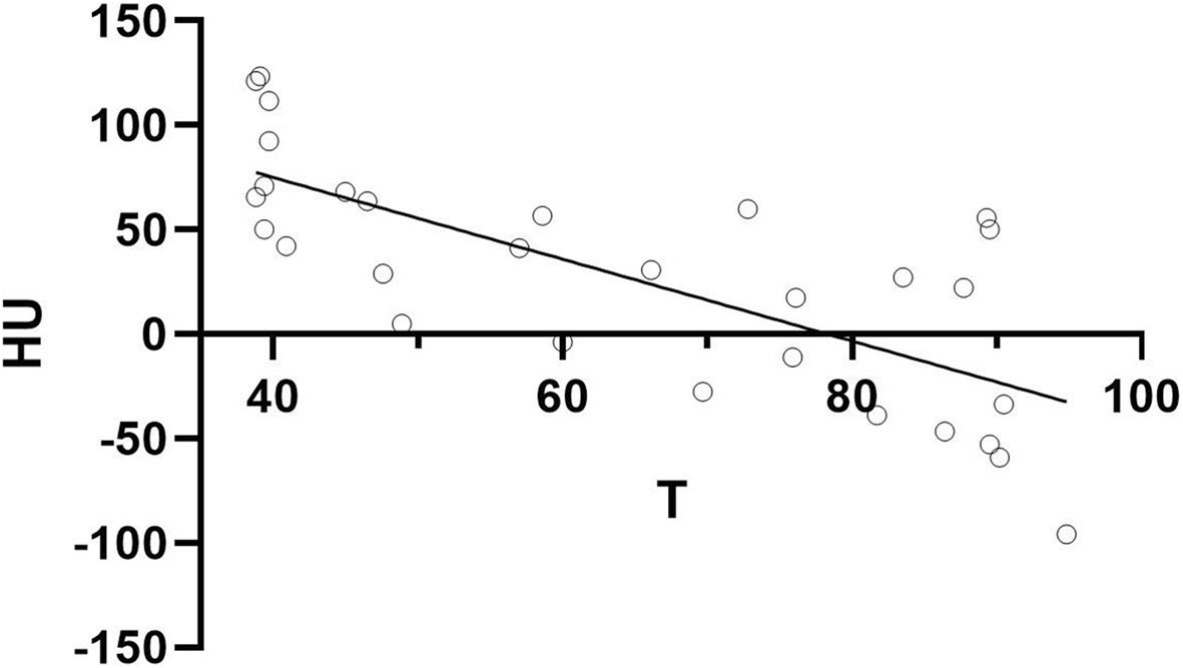
Simple linear regression between temperature and average attenuation in the ablation zone. There is significant correlation between HU and temperature with *r* =  − 0.77 (95% CI, − 0.89 to − 0.57) at *p* < 0.001. Linear regression yields a slope of − 1.96 HU/°C (95% CI, − 2.66 to − 1.26)

**Fig. 3:**
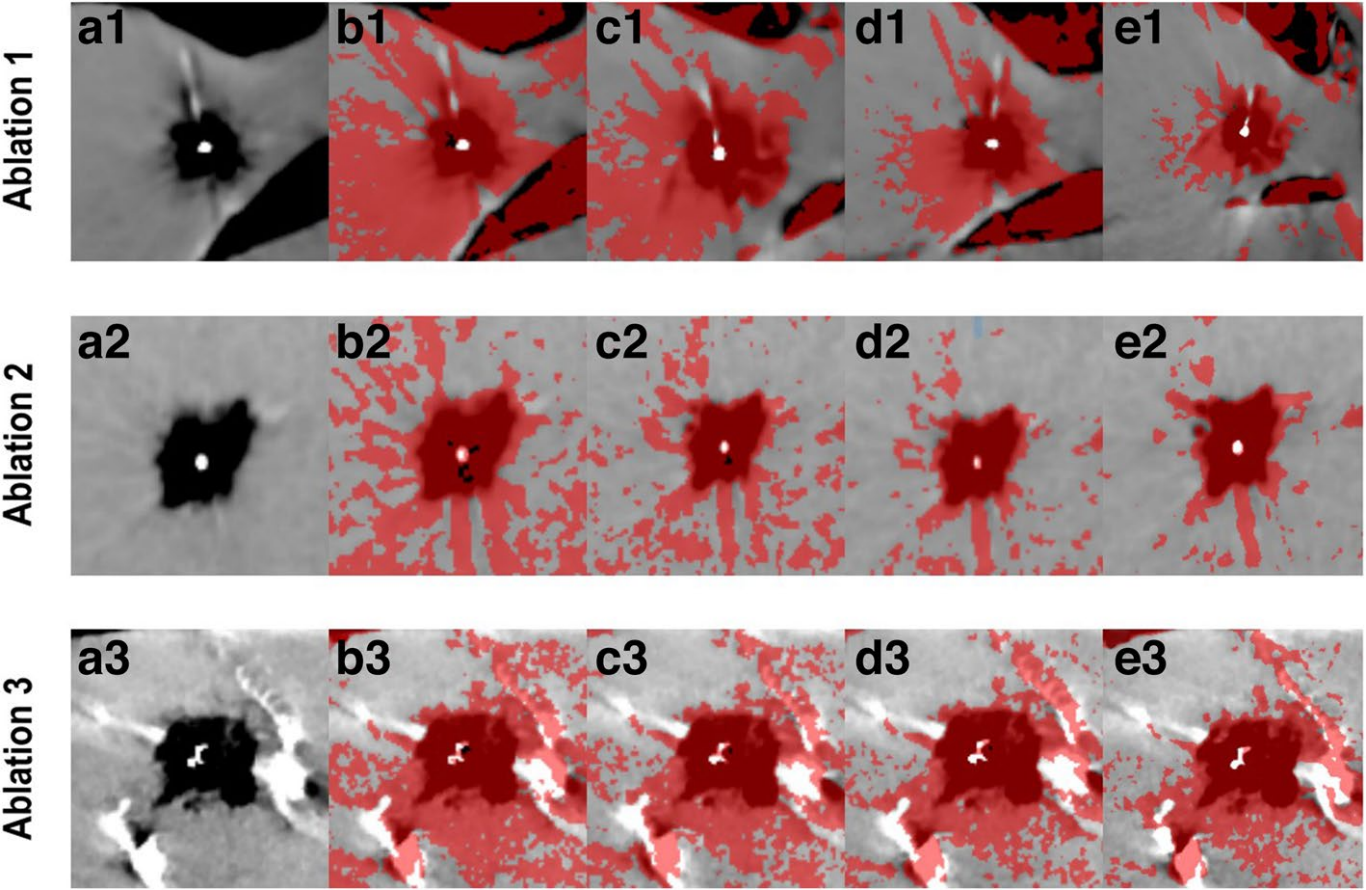
Ablation areas in the pig liver at *T*_max_ (**a**) with corresponding CTT maps (**b–e**). The representation of the ablation zone changes with the use of different slopes based on previous publications [3; 11; 20; 21]. **b** Slope of − 0.5 HU/°C. **c** Slope of − 1.0 HU/°C. **d** Slope of − 1.5 HU/°C. **e** Slope of − 1.96 HU/°C (based on this experiment). Red color represents tissue temperature over 70 °C

For the correlation between tissue attenuation and temperature during the heating phase, a correlation coefficient of *r* =  − 0.77 (95% confidence interval (CI), − 0.89 to − 0.57) with *p* < 0.001 was calculated after confirming non-normal distribution of the data, and linear regression yielded a slope of − 1.96 HU/°C (95% CI, − 2.66 to − 1.26) with *r* ^2^ = 0.54 at *p* < 0.001, which were used by CTT to generate a color map (Fig. 2). Several slopes were used to visualize the CTT mapping spectrum. As slopes were decreased from − 0.5 to − 1.96, artifacts in the periphery were reduced and the ablation zone became more distinct (Fig. 3). The ablation areas determined by using the slope of − 1.96 calculated from our experiment were 760 mm^2^, 240 mm^2^, and 1715 mm^2^ (Fig. 1). Compared with macroscopic assessment, CTT slightly overestimated the areas for ablations 1 and 3, while it underestimated the area for ablation 2 (Fig. 1). CECT overestimated all three areas compared with macroscopy: 892 mm^2^, 542 mm^2^, and 1859 mm^2^ (Fig. 1).

**Fig. 4:**
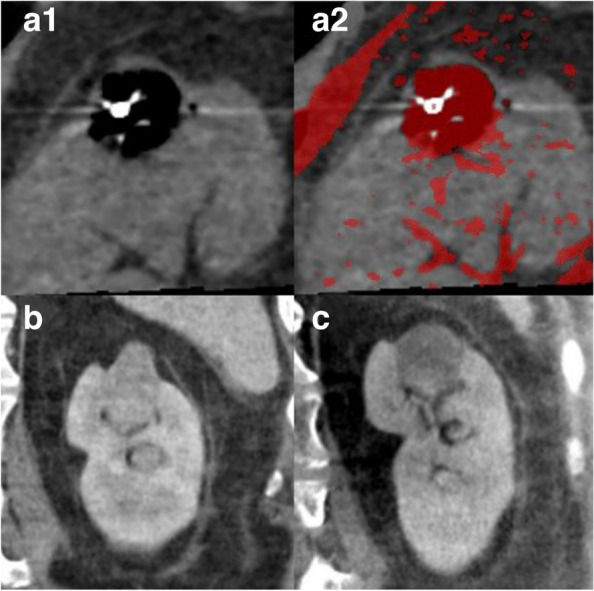
Left kidney of a patient with renal cell carcinoma. The upper panel shows the RFA area at *T*_max_ (**a1**) with the corresponding CTT map (**a2**). The lower panel presents unenhanced CT scans obtained 4 weeks before (**b**) and 5 weeks after ablation treatment (**c**)

The CTT findings retrospectively obtained in the patient who underwent RFA for renal cell carcinoma are presented (Fig. 4). While histopathological reference measurement is not available in this case, the RCC is recognizable on unenhanced CT 4 weeks before ablation (Fig. 4C), and ablated necrotic tissue is apparent in unenhanced CT performed 5 weeks after ablation (Fig. 4D).

## Discussion

### Summary

In this experimental study, we investigated CT thermography in a porcine liver in an in vivo setup. We compared CT thermography with macroscopy and postablation CECT to predict the extent of the ablation zone. Retrospective use of CTT in a patient with RCC who underwent RFA illustrates the feasibility of the method in a clinical setting. Furthermore, CECT overestimated the sizes of the ablation areas due to limitations of the protocol used in our experiment.

### Previous studies

A previous study using subtraction CT for ablation size measurement after MWA has shown strong correlation with histologically determined lesion sizes [18]. However, the authors also emphasize the research gap in determining the ablation area with SCT [18]. Nevertheless, SCT might be used to further calibrate CTT for more precise determination of ablation zone size (Supplementary Figs. 2 and 3). Various research groups have evaluated CT-based thermography in ex vivo and in vivo studies focusing on temperature sensitivity [3, 11, 20, 21]. They investigated a range of slopes, typically rather small ones, using different experimental protocols. The differences in calculated slopes may be due to several factors, including different heating methods, probe power, or duration of heat exposure. In addition, the size and position of the ROIs used for HU determination, the type of experimental setup (in vivo, ex vivo, or in vitro), and the device manufacturers may be potential sources of these discrepancies. The main problem is the correct determination of the HU values, which depends on the ROIs used and the condition of the ablated tissue [3, 11]. However, with a similar experimental protocol and similar devices, a result very close to the slope of − 2.00 HU/°C found in an ex vivo phantom study was achieved [3]. Our results show that using smaller slopes cause artifacts in CTT, which would have made measurement of the ablation zone impossible. While CECT is used to evaluate the outcome of ablation in the clinical setting, the ablation area measured with this method corresponds to the histologically defined red zone, which contains edema and vital cells, thus overestimating the zone with fully destroyed cells [7, 10, 18].

### Limitations

In this experiment, only one pig was used, so the number of ablations was small and limited by the size of the liver. As CTT is still in the development phase, the visualized areas may not be accurate. It is possible that other factors, such as the type of ablation or the type of tissue, need to be considered in the model. In addition, edema formation in the peripheral zone of an ablated area can further confound lesion size measurement. Furthermore, the average HU values may affect the accuracy of our method as well as the slope [3]. Measurements of the same ablation were not repeated to test for consistency. As we monitored ablations with serial CT scans acquired during the downslope phase, there was a 10-min delay, probably resulting in pronounced swelling of the ablated tissue, which we believe also contributed to an overestimation of the ablation zone in CECT. Monitoring of the downslope phase was part of the protocol but proved not to be revealing in this study. The total radiation exposure during the CT examination is increased by the additional image series. However, in the clinical setting, it is important to reduce radiation dose to ensure the safety of both the patient and the interventional radiologist. The planning of the additional image series is targeted, focusing on the tumor tissue and surrounding organs to keep the additional radiation exposure as low as possible. Tissue contraction after organ removal may have affected macroscopic measurement. Retrospective use of CTT in a single patient can merely illustrate the CTT procedure in the clinical setting. RFA was performed in the patient, and data on MWA were not obtained. This study did not evaluate the effects of CTT on patients’ outcome factors such as duration of MWA, complication rates, local recurrence rates, or mortality. Furthermore, our data cannot be directly transferred to human patients or other organs [3, 11]. Gas generation in the upslope phase remains a major problem in CT thermography and could complicate attenuation measurement.

## Conclusions

In this experimental in vivo study, we investigated whether the ablation zone can be predicted using CT-based thermography. A significant correlation of attenuation with temperature was confirmed in our experimental setup. Our results show that CTT allows visualization of the ablation area and measurement of its size and is feasible in patients, encouraging further exploration in a clinical setting.

Intraprocedural monitoring provides valuable feedback on whether tumor margins have been reached or not. This may in the future reduce the rate of local recurrencies and, thus, the number of further invasive procedures. As soon as technical limitations are sufficiently managed, clinical trials are essential to demonstrate the efficacy of intraprocedural monitoring with CTT to test whether its application improves patients’ outcomes.

### Supplementary Information

**Additional file 1:** **Supplementary Figure 1.** Macroscopic images (A) and CECT (B) of three MWAs (1–3) are presented. **Supplementary Figure 2.** Depiction of the ablation zone and its borders in one ablation. A: Unenhanced CT scan at the time of peak temperature (10th scan). B: Subtraction CT (SCT) with multiple ROIs, one line in one direction of a total of eight is shown. ΔHU in SCT approaching zero with increasing distance from the center predicts the average ablation zone border with a radius of *r* = 15 mm. C: Overlay of SCT and macroscopic image. Macroscopic assessment yielded an average radius for this ablation of *r* = 14.5 mm including white and light red zones. The white circle outlines the predicted ablation area, which includes ablated area and barely touches the red zone of the macroscopic image with intercellular edema and vital cells. **Supplementary Figure 3.** ΔHU values obtained by subtraction CT (T0-Tmax). The graph shows pooled data of all sequentially determined ROIs with increasing distance from the MWA probe in ablation 1. Values at less than 8 mm distance to the probe tip are not represented due to artificially high ΔHU values in the center of the ablation, which are attributable to the probe material and gas formation. Dashed vertical line represents the radius determined by macroscopy (14.5 mm).

## Data Availability

The study data are available upon request.

## Abbreviations

CECT: Contrast enhanced computed tomography
CI: Confidence interval
CTDIvol: Computed tomography dose index-volume
CTT: Computed tomography thermography
DLP: Dose-length product
HU: Hounsfield unit
IV: Intravenous
MWA: Microwave ablation
RFA: Radiofrequency ablation
ROI: Region of interest
SCT: Subtraction computed tomography
