# Supplementary material for: Computed tomography-based thermography (CTT) in microwave ablation: prediction of the heat ablation zone in the porcine liver
Source: Insights Imaging. 2023 Nov 14;14:189. doi: 10.1186/s13244-023-01537-z (PMC10645839; doi:10.1186/s13244-023-01537-z)

**Computed tomography-based thermography (CTT) in microwave ablation: prediction of the heat ablation zone in the porcine liver**

**ELECTRONIC SUPPLEMENTARY MATERIAL**

**Supplementary Figure 1**: Macroscopic images (**A**) and CECT (**B**) of three MWAs (**1-3**) are presented.


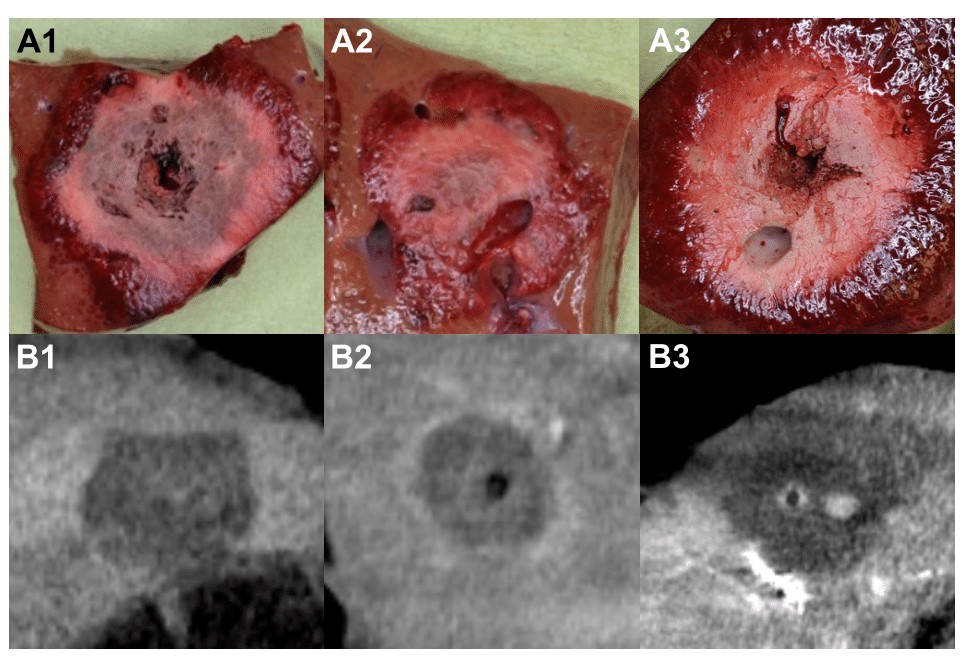


**Supplementary Figure 2**: Depiction of the ablation zone and its borders in one ablation. **A**: Unenhanced CT scan at the time of peak temperature (10^th^ scan). **B**: Subtraction CT (SCT) with multiple ROIs, one line in one direction of a total of eight is shown. ΔHU in SCT approaching zero with increasing distance from the center predicts the average ablation zone border with a radius of r=15 mm. **C**: Overlay of SCT and macroscopic image. Macroscopic assessment yielded an average radius for this ablation of r=14.5 mm including white and light red zones. The white circle outlines the predicted ablation area, which includes ablated area and barely touches the red zone of the macroscopic image with intercellular edema and vital cells.
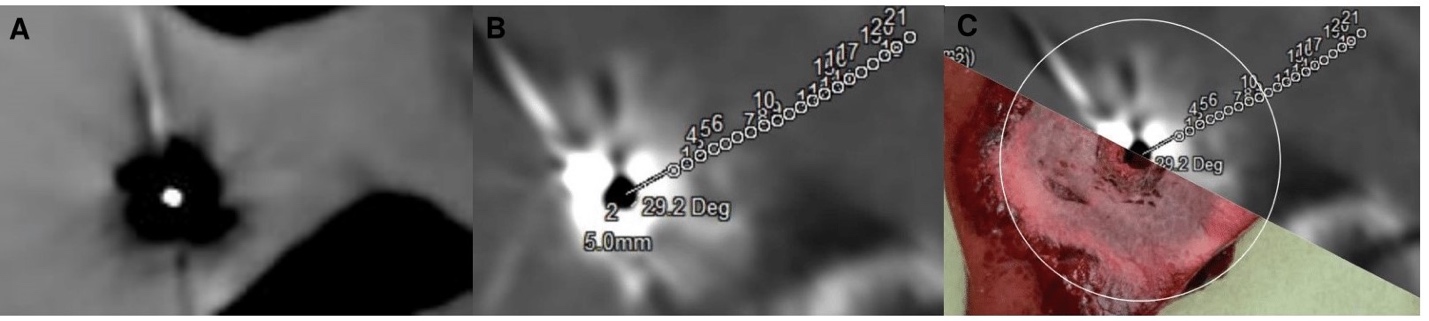


**Supplementary figure 3**: ΔHU values obtained by subtraction CT (T_0_-T_max_). The graph shows pooled data of all sequentially determined ROIs with increasing distance from the MWA probe in ablation 1. Values at less than 8 mm distance to the probe tip are not represented due to artificially high ΔHU values in the center of the ablation, which are attributable to the probe material and gas formation. Dashed vertical line represents the radius determined by macroscopy (14.5 mm).


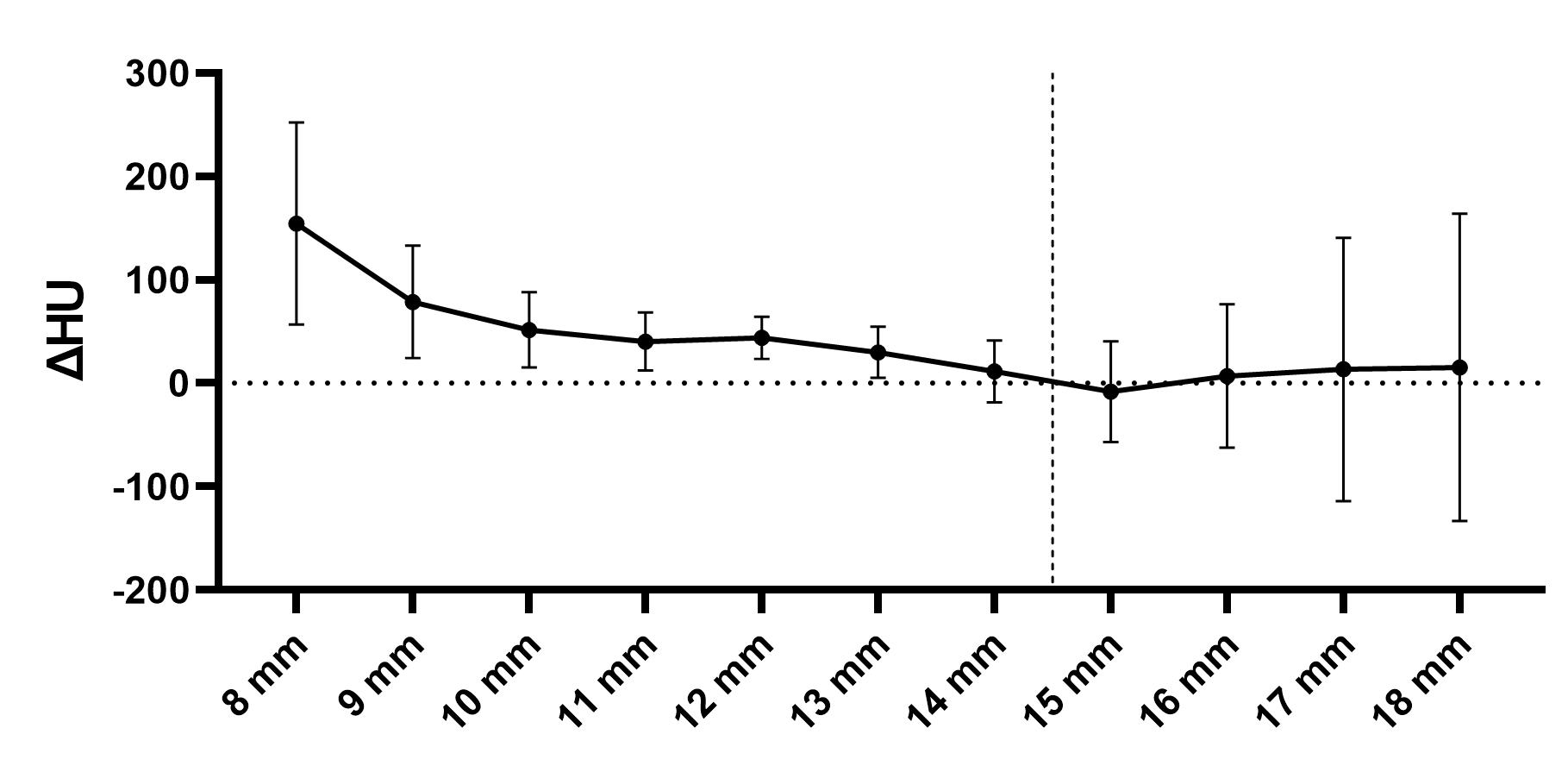

Supplement: Supplementary file 1 — Additional file 1: Supplementary Figure 1. Macroscopic images (A) and CECT (B) of three MWAs (1–3) are presented. Supplementary Figure 2. Depiction of the ablation zone and its borders in one ablation. A: Unenhanced CT scan at the time of peak temperature (10th scan). B: Subtraction CT (SCT) with multiple ROIs, one line in one direction of a total of eight is shown. ΔHU in SCT approaching zero with increasing distance from the center predicts the average ablation zone border with a radius of r = 15 mm. C: Overlay of SCT and macroscopic image. Macroscopic assessment yielded an average radius for this ablation of r = 14.5 mm including white and light red zones. The white circle outlines the predicted ablation area, which includes ablated area and barely touches the red zone of the macroscopic image with intercellular edema and vital cells. Supplementary Figure 3. ΔHU values obtained by subtraction CT (T0-Tmax). The graph shows pooled data of all sequentially determined ROIs with increasing distance from the MWA probe in ablation 1. Values at less than 8 mm distance to the probe tip are not represented due to artificially high ΔHU values in the center of the ablation, which are attributable to the probe material and gas formation. Dashed vertical line represents the radius determined by macroscopy (14.5 mm). [file 13244_2023_1537_MOESM1_ESM.docx]
